# Safety, Immunogenicity, and Protective Efficacy of Intradermal Immunization with Aseptic, Purified, Cryopreserved *Plasmodium falciparum* Sporozoites in Volunteers under Chloroquine Prophylaxis: A Randomized Controlled Trial

**DOI:** 10.4269/ajtmh.15-0621

**Published:** 2016-03-02

**Authors:** Guido J. H. Bastiaens, Maurits P. A. van Meer, Anja Scholzen, Joshua M. Obiero, Mansoureh Vatanshenassan, Tim van Grinsven, B. Kim Lee Sim, Peter F. Billingsley, Eric R. James, Anusha Gunasekera, Else M. Bijker, Geert-Jan van Gemert, Marga van de Vegte-Bolmer, Wouter Graumans, Cornelus C. Hermsen, Quirijn de Mast, André J. A. M. van der Ven, Stephen L. Hoffman, Robert W. Sauerwein

**Affiliations:** Department of Medical Microbiology, Radboud University Medical Center, Nijmegen, The Netherlands; Sanaria Inc., Rockville, Maryland; Department of Internal Medicine, Radboud University Medical Center, Nijmegen, The Netherlands

## Abstract

Immunization of volunteers under chloroquine prophylaxis by bites of *Plasmodium falciparum* sporozoite (PfSPZ)–infected mosquitoes induces > 90% protection against controlled human malaria infection (CHMI). We studied intradermal immunization with cryopreserved, infectious PfSPZ in volunteers taking chloroquine (PfSPZ chemoprophylaxis vaccine [CVac]). Vaccine groups 1 and 3 received 3× monthly immunizations with 7.5 × 10^4^ PfSPZ. Control groups 2 and 4 received normal saline. Groups 1 and 2 underwent CHMI (#1) by mosquito bite 60 days after the third immunization. Groups 3 and 4 were boosted 168 days after the third immunization and underwent CHMI (#2) 137 days later. Vaccinees (11/20, 55%) and controls (6/10, 60%) had the same percentage of mild to moderate solicited adverse events. After CHMI #1, 8/10 vaccinees (group 1) and 5/5 controls (group 2) became parasitemic by microscopy; the two negatives were positive by quantitative real-time polymerase chain reaction (qPCR). After CHMI #2, all vaccinees in group 3 and controls in group 4 were parasitemic by qPCR. Vaccinees showed weak antibody and no detectable cellular immune responses. Intradermal immunization with up to 3 × 10^5^ PfSPZ-CVac was safe, but induced only minimal immune responses and no sterile protection against Pf CHMI.

## Introduction

Malaria accounted for an estimated 198 million clinical cases and 584,000 deaths in 2013, with children under 5 years of age in sub-Saharan Africa most severely affected.^1^ Significant advances have been made in malaria control between 2000 and 2013: an expansion of malaria interventions helped to reduce malaria incidence by 30% globally and by 34% in Africa.^1^ To ensure these positive trends and maintain gains achieved over the past decade, current control and preventive measures such as artemisinin-based combination therapies, rapid diagnostic tests, long-lasting insecticidal nets, and indoor residual spraying should be supported by a highly effective malaria vaccine. Emergence of artemisinin-resistant malaria in southeast Asia^2^,^3^ and widespread insecticide resistance in malaria transmitting anopheline mosquitoes^4^ further increase this need. Combining various control and preventive measures including large-scale vaccination will ultimately offer the best prospect for success.

Progress in the clinical development of efficient immunization strategies as a forerunner of an effective malaria vaccine has been facilitated by controlled human malaria infections (CHMIs). CHMIs involve small groups of malaria-naive volunteers exposed to the bites of *Plasmodium falciparum* sporozoite (PfSPZ)–infected laboratory-reared anopheline mosquitoes. We have previously shown that healthy malaria-naive volunteers can be fully protected against a CHMI by mosquito bite with a homologous Pf strain for more than 2 years after three immunizations under chloroquine prophylaxis by bites from 12 to 15 PfSPZ-infected mosquitoes at monthly intervals (chemoprophylaxis and sporozoites [CPS]).^5^,^6^ Chloroquine kills disease-associated blood stages but does not affect pre-erythrocytic (sporozoite or liver) stages, which are exposed to the host's immune system. CPS-induced protection is mediated by immunity against pre-erythrocytic stages.^7^

Although being a strong proof of concept, this protocol is unsuitable for direct practical application as long as PfSPZ are inoculated by mosquito bites. Sanaria Inc. (Rockville, MD) has developed a process for manufacturing infectious, aseptic, purified, vialed, and cryopreserved PfSPZ (Sanaria^®^ PfSPZ Challenge).^8^–^13^ To date, single doses of cryopreserved PfSPZ have been administered at different doses up to 1.25 × 10^5^ PfSPZ in 221 human subjects by the intradermal (ID) (*N* = 84), intramuscular (IM) (*N* = 70), intravenous (IV), or direct venous inoculation (DVI) (*N* = 67) routes using a needle and syringe to assess safety, tolerability, and infectivity.^8^,^10^–^13^

Here, we report the first phase I/IIb trial of CPS immunization with aseptic, purified, and cryopreserved PfSPZ, an approach called PfSPZ-CVac (PfSPZ-chemoprophylaxis vaccine) to assess safety, tolerability, immunogenicity, and protection against a standard homologous CHMI with five PfSPZ-infected mosquitoes.

## Materials and Methods

### Study population.

We recruited healthy male and female subjects aged 18 to 35 years without a history of malaria, adhering to inclusion and exclusion criteria as described previously.^7^ All subjects had an estimated 10-year risk of developing a cardiac event of less than 5% as estimated by the systematic coronary evaluation system.^14^ Baseline ophthalmologic examination revealed no abnormalities on fundoscopy that might preclude treatment with chloroquine.

Subjects gave written informed consent before inclusion. The trial was conducted in accordance with Good Clinical Practice and approved by the Central Committee for Research Involving Human Subjects of The Netherlands (CCMO NL39541.091.12). An Investigational New Drug application was filed with the U.S. Food and Drug Administration; Clinicaltrials.gov identifier: NCT01728701.

### Trial design.

**Figure 1.**
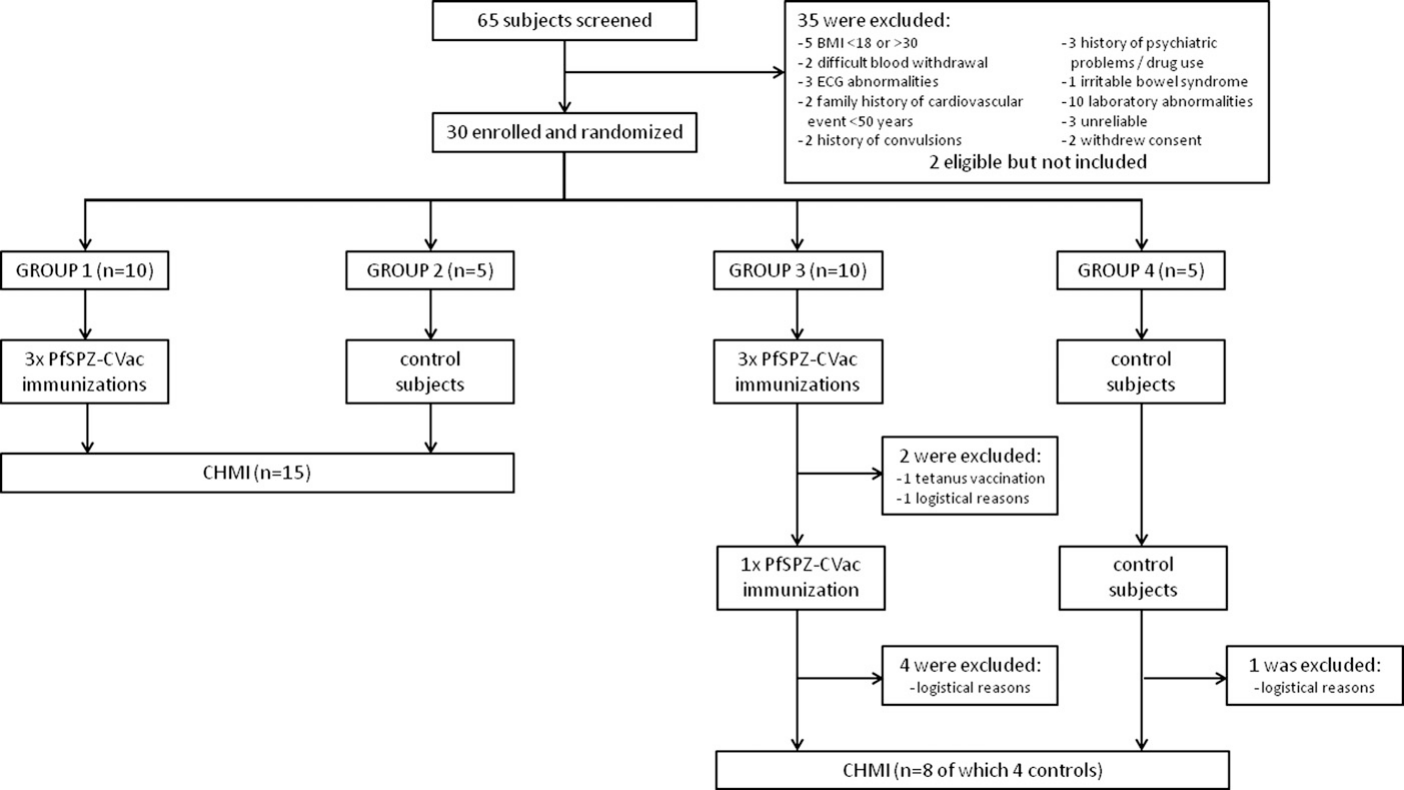
Trial flow chart. On days 8, 36, and 64 after initiation of chloroquine chemoprophylaxis, vaccine groups 1 and 3 received injections containing 7.5 × 10_4_ *Plasmodium falciparum* sporozoites (PfSPZ), while control groups 2 and 4 received normal saline. On day 124, groups 1 and 2 underwent controlled human malaria infection (CHMI) with PfSPZ by mosquito bite. Groups 3 and 4 received additional PfSPZ injections on day 232 and underwent CHMI on day 369. BMI = body mass index; ECG = electrocardiogram.

This prospective, single center, double-blind, randomized, placebo-controlled clinical trial was performed at the Radboud University Medical Center (Radboudumc), Nijmegen, The Netherlands, from September 2012 to February 2014. Thirty subjects were randomly assigned to four study groups: vaccine groups 1 and 3 (each 10 subjects) and control groups 2 and 4 (each five subjects) (Figure 1

). All groups received ID injections with either aseptic, purified, cryopreserved, and infectious PfSPZ (PfSPZ Challenge)^8^ or normal saline (NS) under chloroquine cover as described below. Sixty days after the last immunization with PfSPZ Challenge, groups 1 and 2 received a standard CHMI by five mosquitoes infected with Pf NF54 SPZ.^15^ Protection was defined as thick smear negative through day 21 post-CHMI. Subsequent study procedures involving groups 3 and 4 were dependent on the rate of protection: if ≥ 75%, groups 3 and 4 would receive CHMI with heterologous Pf NF135.C10-infected mosquitoes^16^; if < 75%, groups 3 and 4 would receive a fourth PfSPZ-CVac immunization or NS injection, respectively, followed by homologous Pf NF54 CHMI.

### PfSPZ-CVac immunizations.

All subjects received standard chloroquine chemoprophylaxis for a period of 13 weeks (91 days) as described previously.^5^ Of chloroquine base, 300 mg was given on days 0, 1, and 7 and weekly thereafter through to day 91. On days 8, 36, and 64, vaccine groups received six ID 10 μL injections (three injections in the deltoid region of each arm) of PfSPZ Challenge, containing a total of 7.5 × 10^4^ PfSPZ. Controls received six ID 10 μL injections of NS in a similar manner as the vaccine groups. Vials of PfSPZ Challenge stored in liquid nitrogen vapor phase were thawed and diluted in phosphate-buffered saline containing 1% human serum albumin, and all subjects were injected within 30 minutes of thawing. Because the protection threshold was not met after the CHMI administered to groups 1 and 2, subjects in groups 3 and 4 received a fourth injection of PfSPZ Challenge or NS, 168 days after the third immunization.

On days 5 and 10–14 after injections, subjects were checked on an outpatient basis by attending physicians and blood was drawn for thick blood smears; standard hematological (full blood count, platelets and differentiation of white blood cells) and biochemical (sodium, potassium, creatinine, urea nitrogen, aspartate aminotransferase, alanine aminotransferase, alkaline phosphatase, γ-glutamyl transferase, and total bilirubin) parameters; markers of myocardial tissue damage, coagulation, inflammation, and hemolysis as described previously (highly sensitive troponin T, D-dimer, and lactate dehydrogenase)^7^; and retrospective assessment of blood-stage parasitemia by quantitative real-time polymerase chain reaction (qPCR). Additional blood samples for qPCR measurements were provided on a voluntary basis on days 8 and 9 after the second and third immunizations. All signs and symptoms (solicited and unsolicited) were recorded and graded as follows: mild/grade 1 (awareness of symptoms that were easily tolerated and did not interfere with usual daily activity), moderate/grade 2 (discomfort that interfered with or limited usual daily activity), or severe/grade 3 (disabling, with subsequent inability to perform usual daily activity, resulting in absence or required bed rest). Tympanic temperature was measured and recorded as fever grade 1 (37.6–38.0°C), grade 2 (> 38.0–39.0°C), or grade 3 (> 39.0°C). Causality of adverse events (AEs) was classified as not, possibly, probably, or definitely related to the trial.

### CHMI by mosquito bite.

*Anopheles stephensi* mosquitoes were reared at the Radboudumc insectary and infected by feeding on cultured gametocytes of Pf N54 parasites, according to standard procedures as described previously.^17^ Of the mosquitoes used for CHMI, 100% had PfSPZs in their salivary glands, and mosquitoes were infected with an average of 75,800 and 98,000 PfSPZs per mosquito for CHMI #1 (groups 1 and 2) and CHMI #2 (groups 3 and 4), respectively.

Thirty-three days after the last dose of chloroquine, corresponding to 60 days after the last immunization with cryopreserved PfSPZ, vaccine group 1 (*N* = 10) and control group 2 (*N* = 5) underwent CHMI by allowing five *An*. *stephensi* sporozoite–infected mosquitoes to feed for 10 minutes as described previously.^15^ The salivary glands of all blood-engorged mosquitoes were dissected to confirm the presence of PfSPZs. When necessary, feeding sessions were repeated with fewer mosquitoes until exactly five infectious mosquitoes had fed. Starting from day 5 after CHMI, subjects were checked daily on an outpatient basis as described above for PfSPZ-CVac immunizations. Blood sampling for thick smear reading and retrospective assessment of parasitemia by qPCR was performed once daily on days 5 and 6, twice daily on days 7–15, once daily on days 16–21, and for 2 days after initiation of antimalarial treatment. Antimalarial treatment, consisting of a curative regimen of atovaquone/proguanil (1,000/400 mg) once daily for 3 days, was initiated either as soon as parasites were detected on a thick blood smear or 21 days after CHMI by mosquito bite for those who did not become infected. Final follow-up visits were on days 35 and 140 after CHMI.

On day 14 after CHMI, one subject in vaccine group 1 was unblinded due to a cardiac serious AE (SAE).^18^ All other subjects in groups 1 and 2 were unblinded according to protocol 24 days after CHMI by mosquito bite.

Because of the cardiac SAE, the trial was put on hold for 64 days (March 13, 2013 to May 16, 2013) by the Safety Monitoring Committee and the Central Committee for Research Involving Human Subjects of The Netherlands. New safety measures were adopted for follow-up after mosquito CHMI of groups 3 and 4. The endpoint of thick blood smear positivity for diagnosis of malaria was changed to qPCR positivity. Atovaquone/proguanil treatment was to be initiated after 1) two consecutive positive qPCRs when temperature < 38.0°C, 2) one positive qPCR in the presence of a temperature ≥ 38.0°C, or 3) a positive thick smear prepared upon clinical indication during an evening visit, which took place during days 7–15. Subjects who underwent standard vaccinations within 3 months before start of the trial or were planning to take standard vaccinations during the trial period up to 8 weeks after CHMI were excluded; this is because the study subject with the SAE had received diphtheria, poliomyelitis, tetanus, parenteral typhoid fever, and hepatitis A and B vaccinations between the immunization period and CHMI, and it is possible that these immunizations may have played a role in the myocarditis.^18^

### Study outcome parameters.

The primary study outcome was frequency and magnitude of AEs. Secondary study outcomes included occurrence of Pf parasitemia after each immunization and CHMI, as assessed by microscopic examination of thick blood smears and/or qPCR. Thick blood smears were prepared and read as described previously.^7^ qPCR was performed as described previously^19^ with some modifications. In brief, 5 μL Zap-oglobin II Lytic Reagent (ref. no: 7501369-HA; Beckman Coulter, Brea, CA) was added to each 0.5 mL blood sample, mixed and stored at −80°C. After thawing samples were spiked with phocine herpes virus (PhHV) as extraction control and DNA was extracted by a MagnaPure LC isolation instrument (Roche, Basel, Switzerland). Isolated DNA was resuspended in 50 μL H_2_O and 5μL was used as template. For the detection of Pf, the TaqMan MGB probe AAC AAT TGG AGG GCA AG-FAM was used (Thermo Fisher Scientific, Waltham, MA). For the quantification of PhHV, we used primers and probe as described previously.^20^ The sensitivity of qPCR was 35 parasites/mL of whole blood. The prepatent period (by qPCR or thick smear) was defined as the period between mosquito bite CHMI and the first positive qPCR (≥ 500 parasites/mL) or thick smear result (≥ 2 unambiguous parasites). The difference in the prepatent period assessed by qPCR compared with thick smear was defined as Δ prepatency.

### Immunologic analysis.

Plasma and peripheral blood mononuclear cells (PBMCs) were obtained from blood collected into citrated BD (Franklin Lakes, NJ) Vacutainer CPT Cell Preparation Tubes on the following time points: one day before initiation of chloroquine prophylaxis (I1 − 1) for all volunteers, the day before the third immunization (I3 − 1) and CHMI (C − 1, 59 days after the third immunization) in groups 1 and 2, and one day before and 52 days after the fourth injection of PfSPZ/NS injections in groups 3 and 4 (I4 − 1, I4 + 52).

### Analysis of antibody responses by enzyme-linked immunosorbent assay.

Plasma concentrations of malaria antigen-specific antibodies were determined against a pool of 100 sera from adults living in a highly endemic area in Tanzania (HIT serum)^5^ by standardized enzyme-linked immunosorbent assay (ELISA). Recombinant proteins of circumsporozoite protein (PfCSP) and liver stage antigen 1 (PfLSA-1)^21^ are expressed by sporozoites and liver stages, respectively, while merozoite surface protein 1 recombinant protein (PfMSP-1)^22^ is expressed by late liver and blood stages.^23^ Antibody reactivity to these antigens was determined to assess exposure to and thus induction of immunity to the different stages of the malaria cycle during the immunization regimen.

The 96-well polystyrene flat-bottom plates (NUNC^™^ Maxisorp; Thermo Fisher Scientific) were coated overnight at 4°C with 2 μg/mL of antigen, washed with phosphate-buffered saline (PBS) and blocked for 1.5 hours at room temperature (RT) with 150 μL of 5% milk in PBS. In all the washing steps that followed, plates were washed with PBS + 0.05% Tween (PBST). Serially diluted plasma samples (starting at 1:50 to 1:800 in 1% milk in PBST [sample buffer]) were incubated for 3 hours at RT in a humidified chamber. As a standard, duplicates of pooled HIT serum were included on every plate in a 7-point dilution series. Reactivity for each antigen in undiluted HIT serum was defined as 100 arbitrary units (AU). Bound immunoglobulin G (IgG) was detected using horseradish peroxidase (HRP)–conjugated antihuman IgG (Thermo Fisher Scientific) followed by TMB One Component HRP Microwell Substrate (Tebu Bio, Heerhugowaard, The Netherlands). The reaction was stopped using 0.2 M H_2_SO_4_ and absorbance was measured with a spectrophotometer plate reader at 450 nm (Anthos 2020 ELISA plate reader, Cambridge, UK). Optical density values were converted into AUs by four-parameter logistic curve fit using Auditable Data Analysis and Management System for ELISA (ADAMSEL v1.1; http://www.malariaresearch.eu/content/software).

### Antibodies against PfSPZs by immunofluorescense assay.

Aseptic, purified PfSPZ suspended at 2 × 10^3^ in 20 μL PBS with 2% bovine serum albumin were added to Cel-Line (Thermo Fisher Scientific) immunofluorescense assay (IFA) slides as described before.^24^ Pre-immune control sera (I1 − 1) were added at a single dilution of 1:50; the post-immune samples of 52 days after the fourth injection (I4 + 52) were added at 2-fold dilutions starting at 1:50. Anti-PfCSP monoclonal antibody 2A10^25^ was used as positive control. After incubation at 37°C for 1 hour, slides were washed and Alexa fluor 488–conjugated goat antihuman IgG (cat. no. A11013; Molecular Probes, Thermo Fisher Scientific) (1:250 in 0.2% Evans blue) was added followed by incubation at 37°C for 1 hour and a washing step. Vectashield mounting medium (Vector Laboratories, Burlingame, CA) was added to each well, and a cover slip placed on the slide. Samples were assessed with an Olympus (Shinjuku, Tokyo, Japan) BX51 fluorescence microscope at ×400 magnification. The positive control was a serum specimen from a volunteer immunized with PfSPZ vaccine (radiation-attenuated PfSPZ) and a malaria-naive serum sample was used as negative control.^24^ The endpoint titer was defined as the last serum dilution at which fluorescence intensity was higher than pre-immune sera. A postimmunization serum sample was considered positive if it had fluorescence at a dilution of 1:50 or higher, and the preimmunization serum from that volunteer was negative at 1:50.

### Analysis of cellular immune responses by flow cytometry.

For the assessment of Pf-specific immune responses, in vitro restimulation assays of PBMCs were performed as described previously.^26^ In brief, cryopreserved PBMCs were thawed and stimulated in vitro for 24 hours with glycerol-cryopreserved schizont-stage Pf NF54–infected erythrocytes (PfRBCs) and aseptic, purified, cryopreserved Pf NF54 SPZ prepared like PfSPZ Challenge in the presence of antihuman CD107a antibody (Pacific Blue, H4A3, Biolegend, San Diego, CA) at 100 μL/well (final concentrations: 5 × 10^6^ PBMC/mL; 10 × 10^6^ PfRBC/well; 1.25 × 10^6^ PfSPZ/mL). Uninfected red blood cells (uRBCs) and medium with 1% HSA (AlbuRx 25; CSL Behring AG, Bern, Switzerland) were used as a negative control. For the last 4 hours, Brefeldin A (10 μg/mL; Sigma-Aldrich, St. Louis, MO) and Monensin (2 μM; Sigma) were added, along with PMA (50 ng/mL; Sigma) and ionomycin (1 μg/mL; Sigma) in positive control wells. Cells were stained with a viability marker (live/dead fixable dead cell stain aqua; Invitrogen, Carlsbad, CA) and antibodies against CD3 (PerCp, UCHT1), interferon gamma (IFN-γ [PECy7, 4S.B3]), and granzyme B (FITC, GB11; all Biolegend); CD4 (ECD, SFCI12T4D11), γΔT cell receptor (PE, IMMU510; both Beckman Coulter), CD8 (APC-H7, SK1; BD Biosciences) and CD56 (biotin, MEM188 with eBioscience Streptavidin eFluor 660; eBioscience, San Diego, CA).^26^ Intracellular cytokine staining was performed using the Fixation and Permeabilization Buffer Kit (eBioscience). For every individual subject, samples from all time points were thawed, stimulated, and stained within the same experimental round. Flow cytometry was performed on a 9-color Cyan ADP (Beckman Coulter) and data were analyzed using FlowJo software (version 9.6.4; Tree Star, Ashland, OR). Gating of cytokine-positive cells was performed automatically, based on the geometric mean fluorescent intensity of cytokine-negative PBMCs for each subject, time point, and stimulus. Responses to uRBCs were subtracted from the response to PfRBCs for every subject on every time point.

### Analysis of chloroquine and monodesethylchloroquine concentrations.

Plasma of volunteers in groups 1 and 2 collected on the day before CHMI (C − 1) was used to assess chloroquine and monodesethylchloroquine levels. The plasma samples (100 μL) were precipitated by methanol (400 μL) containing hydroxychloroquine (50 ng/mL). After mixing and centrifugation, supernatants were diluted to half in ammonium formate solution 20 mmol/L with formic acid (0.5% v/v), and 15 μL per sample was injected into the system. Chloroquine and monodesethylchloroquine were separated and quantified by liquid chromatography mass detection (TSQ Quantum Ultra; ThermoFisher, France) using an Atlantis DC18 (100 × 2.1 mm, 3 μm) column (Waters, France) using water/methanol (95/5% v/v) with formic acid (0.1% v/v) as mobile phase. The flow rate was 0.30 mL/minute and the column temperature was kept at 25°C. Hydroxychloroquine was used as internal standard. Data were acquired in the positive ion mode with an electrospray ionization source. Multiple reaction monitoring was used for data collection.

### Statistical methods.

Statistical analyses were performed with GraphPad Prism 5 (La Jolla, CA). The difference in AEs between two groups was calculated by unpaired Student *t* test on the cumulative duration of AEs. Differences between groups in prepatent periods by qPCR, in prepatency between thick smear and qPCR (Δ prepatency), and in antibody levels were tested by the Mann–Whitney test. Differences in antibody concentrations between time points within a group were tested by Wilcoxon signed-rank test.

## Results

### Trial overview.

Of 65 screened subjects (median age = 21 years; range = 18–27 years), 30 were included in the study (Figure 1 and Table 1). Of these subjects under chloroquine chemoprophylaxis (groups 1 and 3), 20 were immunized as a single cohort by ID injection three times at 4-week intervals with 7.5 × 10^4^ cryopreserved PfSPZ, while 10 controls (groups 2 and 4) received chloroquine chemoprophylaxis and ID injections of NS following the same schedule. The 15 volunteers in groups 1 and 2 underwent CHMI #1 60 days after the last immunization, corresponding to 33 days after the last chloroquine dose. One subject in vaccine group 3 received tetanus vaccination after the third immunization session and was later excluded based on the safety procedures adopted for CHMI #2, because of the SAE after CHMI #1. Another subject in group 3 was unable to continue participation for the fourth immunization because of logistical reasons. Therefore, 13/15 subjects (8/10 in group 3; 5/5 in group 4) received a fourth immunization of 7.5 × 10^4^ PfSPZ at 168 days after the third immunization (Figure 1). Five of these 13 subjects were not able to participate in CHMI #2 for logistical reasons. Altogether four subjects in each group underwent CHMI #2 at 109 days after the last (fourth) chloroquine dose, corresponding to 137 days after their last injection of PfSPZ or NS.

### Safety and tolerability during PfSPZ-CVac immunizations.

Immunizations were well tolerated. There were no signs of local reactogenicity in vaccinees or controls. Of 20 PfSPZ-CVac recipients, 11 (55%; seven in group 1 and four in group 3) reported predominantly mild to moderate probably/possibly associated solicited AEs (mean duration = 0.3 ± 0.5 days) beginning on days 1–33 after the first three immunizations (Table 2). Six of 10 (60%) NS recipients (three in group 2 and three in group 4) reported predominantly mild to moderate probably/possibly associated solicited AEs (mean duration = 0.3 ± 0.4 days) beginning on days 1 to 20 after the first three immunizations. The other nine PfSPZ-CVac and four NS-inoculated volunteers did not report any complaints. There was no difference in the cumulative duration of probably/possibly related solicited AEs per subject between vaccinees (groups 1 and 3) and controls (groups 2 and 4) (*P* = 0.52). There were no solicited AEs after the fourth PfSPZ-CVac injection in vaccine group 3, and one mild headache in control group 4. Overall, the most commonly reported AE was headache (6/20 [30%] vaccine subjects and 3/10 [30%] controls), which occurred once in a vaccine group 3 volunteer as the single reported grade 3 AE.

After the first PfSPZ-CVac immunization, a remarkable unsolicited AE occurred in one subject in vaccine group 3. Several hours after the fourth chloroquine dose, transitory urticaria developed at multiple sites of the body lasting for 3 days (corresponding to days 5–8 after PfSPZ Challenge injection). The subject did not receive any treatment of the urticaria. This subject had a raised D-dimer level 2 days after resolution of the urticaria (1,060 ng/mL, upper limits of normal being 500 ng/mL) that decreased to 520 ng/mL within the next 4 days. The volunteer continued in the study, received two more immunizations with PfSPZ-CVac and underwent CHMI, but did not develop urticaria or any other indication of an allergic reaction. However, the D-dimer levels were elevated after each of the three following immunizations (range = 520–1,350 ng/mL).

None of the 20 PfSPZ-CVac recipients developed parasitemia during the immunization period, as detected by thick blood smears and retrospectively by qPCR. Furthermore, lymphocyte and platelet counts did not decline after immunizations.

### Protective efficacy after CHMI by Pf-infected mosquitoes.

**Figure 2.**
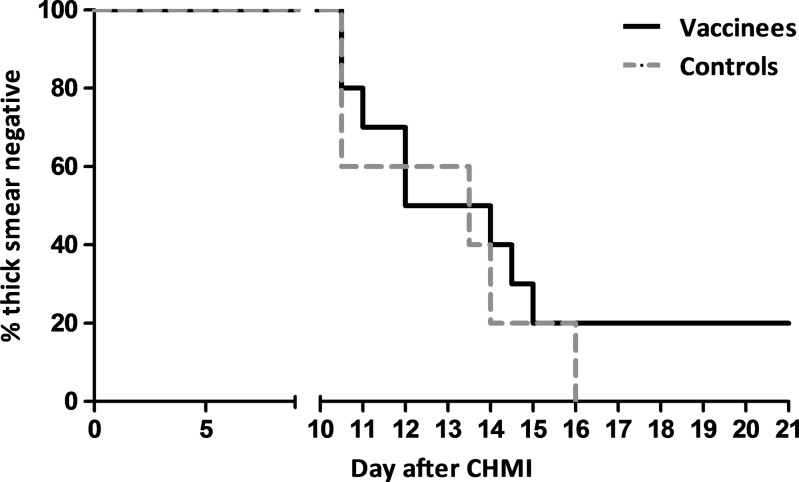
Time to parasitemia by microscopy after controlled human malaria infection (CHMI) #1. The time to thick smear positivity is shown for 10 *Plasmodium falciparum* sporozoite chemoprophylaxis vaccine (PfSPZ-CVac) recipients (group 1, black line) and five control subjects (group 2, gray dashed line).

**Figure 3.**
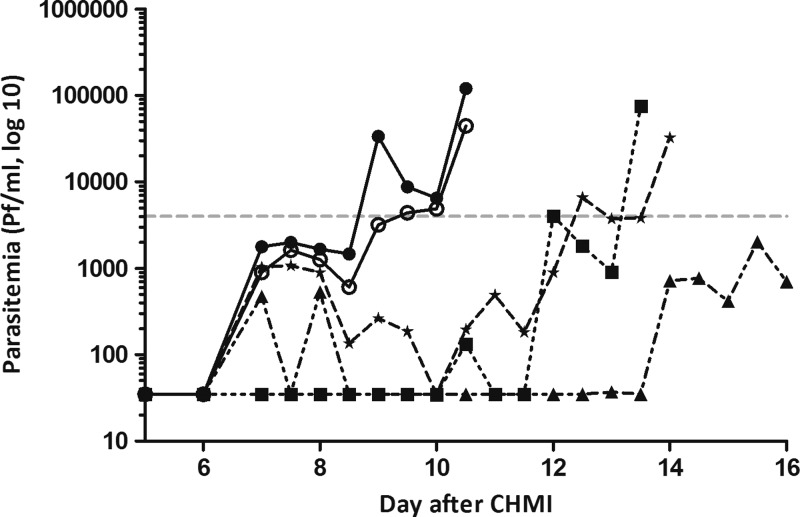
Parasite dynamics by quantitative real-time polymerase chain reaction (qPCR) after controlled human malaria infection (CHMI) #1. Individual parasite density curves of control subjects (*N* = 5) measured by qPCR are shown up to day of treatment, based on diagnosis by thick smear. The gray dotted line indicates the average parasite detection limit for microscopy. Four of five subjects were first positive on day 7. One subject was first positive on day 10.5.

All five controls in group 2 became thick smear positive. However, this group showed a wide variation in prepatent periods (median = 13.5 days, range = 10.5–16 days; Figure 2

and Table 1) and a median Δ prepatency (difference between prepatent period by thick blood smear and qPCR) of 6.5 days (range = 3.0–9.0 days), which was significantly longer compared with previous studies^7^,^26^,^27^ (*P* = 0.006). This wide range is explained by the prolonged parasitemia below the detection limit for microscopy in two of the control subjects (represented by the triangle and star lines in Figure 3

). Retrospective parasitemia measurement by qPCR revealed a median prepatent period of 7.0 days (range = 7.0–10.5 days), comparable to previous studies^7^,^26^,^27^ (*P* = 0.56).

Eight of 10 vaccinees developed patent parasitemia by thick smear (median prepatent period by thick smear of 12 days, range = 10.5–15 days), while two subjects in group 1 remained thick smear negative throughout the 21-day follow-up period (Figure 2). Importantly, both thick smear–negative subjects had positive qPCRs on either day 7 post-CHMI (85 parasites/mL) or days 7 and 7.5 (252 and 265 parasites/mL), but then remained qPCR negative through day 21 post-CHMI (Supplemental Figure 1). This initial prepatent period by qPCR was in line with the other volunteers in this group (range = 7.0–10.5 days) and similar to control group 2 (*P* = 0.55). On the day before CHMI (C − 1), plasma chloroquine was deemed to be below the minimum therapeutic concentration in vivo in all 15 subjects.^28^ However, it is noteworthy that both thick smear–negative subjects had higher levels of chloroquine in plasma (13 μg/L) than all of the thick smear–positive subjects (≤ 5 μg/L). The monodesethylchloroquine levels (principal active metabolite of chloroquine) were not different in these two individuals with negative thick smears than the subjects who were thick smear positive (< 5 μg/L). The combined data suggest that a parasite killing effect of residual chloroquine levels cannot be excluded in the two thick smear–negative but qPCR-positive individuals.

**Figure 4.**
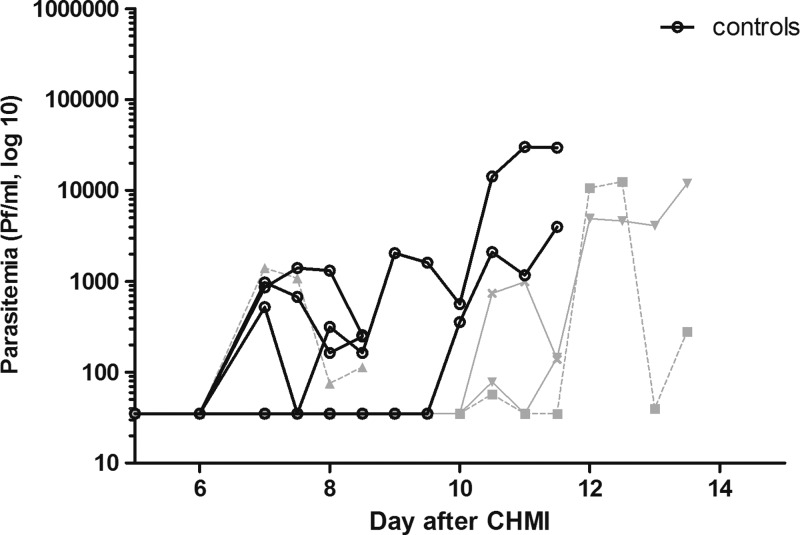
Parasite dynamics by quantitative real-time polymerase chain reaction (qPCR) after controlled human malaria infection (CHMI) #2. Parasite densities are shown until the day of treatment, based on diagnosis by quantitative polymerase chain reaction. Each line represents a single subject: the gray lines represent subjects of vaccine group 3 (*N* = 4), the black lines subjects of control group 4 (*N* = 4).

The second CHMI was administered to groups 3 and 4 after a fourth immunization. All eight subjects became qPCR positive (Figure 4

) with a median prepatent period of 10.5 days (range = 7–10.5 days) in immunized volunteers (*N* = 4) and 7 days (range = 7–10 days) in controls (*N* = 4; *P* = 0.11, Mann–Whitney *U* test, two-tailed). There were no thick smear results to report because all subjects were treated based on qPCR results.

### AEs after CHMI by Pf-infected mosquitoes.

All subjects in groups 1 and 2 experienced solicited AEs possibly or probably related to CHMI (mean number of AEs per subject in group 1 = 6.9, mean duration = 0.6 ± 1.2 days; group 2 = 8.4, mean duration = 0.6 ± 0.7 days), with headache (*N* = 29), fever (*N* = 20), and nausea (*N* = 19) most commonly reported (Table 3). There was no significant difference between the cumulative duration of AEs per subject in group 1 compared with group 2 (3.8 versus 4.7 days, respectively; *P* = 0.64). One SAE occurred in a subject in vaccine group 1 on day 13 after CHMI and 2 days after initiation of treatment with atovaquone/proguanil (72 days after last PfSPZ-CVac immunization), which was diagnosed as acute myocarditis.^18^ Abnormal laboratory values normalized without complications in all subjects.

All volunteers in group 3, except for one, experienced solicited AEs possibly or probably related to CHMI (mean number of AEs per subject in group 3 = 4.8, mean duration = 0.5 ± 0.6 days; in group 4 = 6.8, mean duration = 0.9 ± 1.9 days), with headache (*N* = 15), nausea (*N* = 9), and chills (*N* = 7) as most common symptoms (Table 3). The cumulative duration of AEs per subject in group 3 was similar to group 4 (2.3 versus 6.0 days, respectively; *P* = 0.051). In addition, there was no significant difference between control groups 2 and 4 (*P* = 0.51), although there was a trend toward a lower frequency of AEs in group 4 in which antimalarial treatment was initiated after qPCR instead of thick smear positivity.

### Humoral and cellular immune responses.

**Figure 5.**
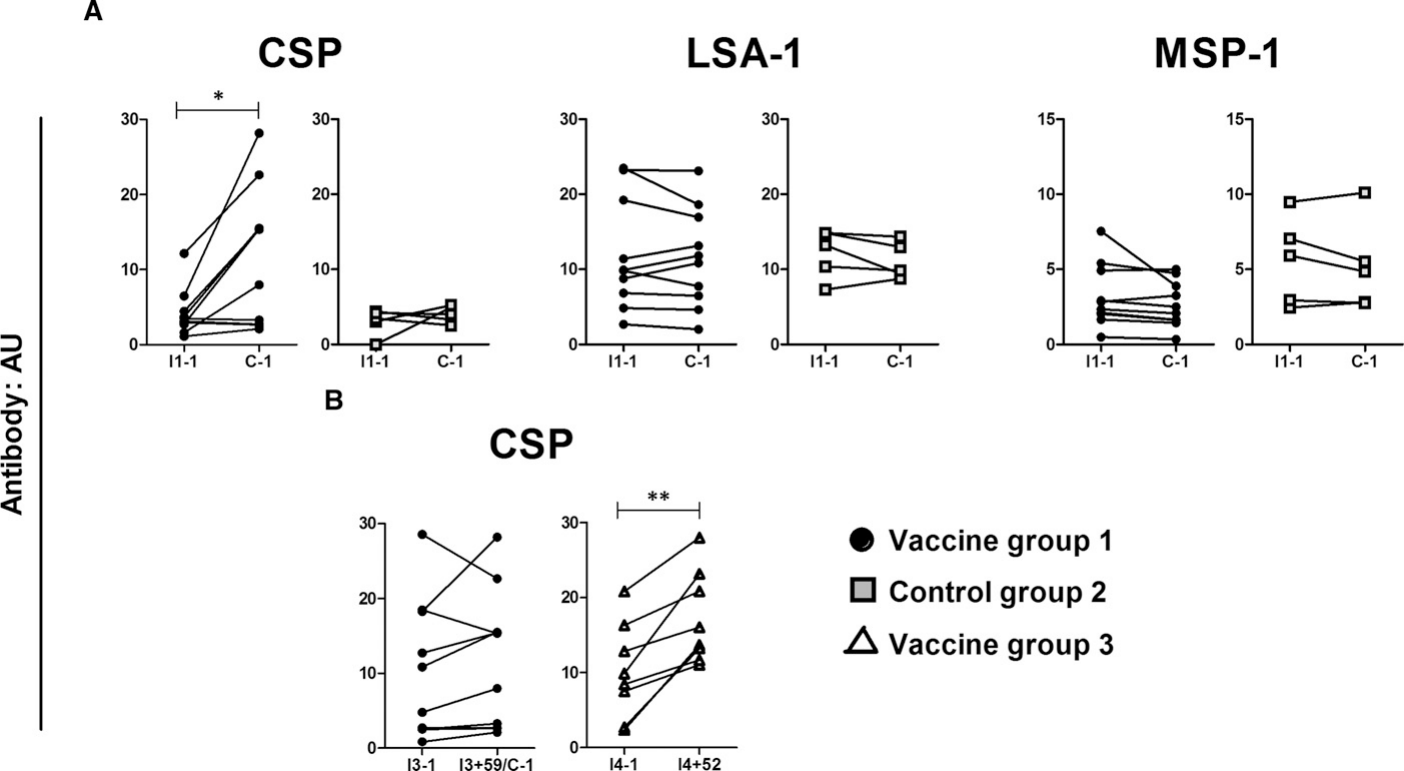
Specific antibody responses induced by immunization by *Plasmodium falciparum* sporozoite chemoprophylaxis vaccine (PfSPZ-CVac). Parasite-specific plasma antibody responses are shown for vaccine group 1 (*N* = 10, black circles), control group 2 (*N* = 5, gray squares), and vaccine group 3 (*N* = 8, white triangles) at the following time points: (**A**) 1 day before the first immunization (I1 − 1) and 59 days after the third immunization (1 day before controlled human malaria infection [CHMI]) (C − 1); (**B**) 1 day before (I3 − 1) and 59 days after the third immunization (I3 + 59) (1 day before CHMI) (C − 1) as well as 1 day before the fourth immunization (I4 − 1) and 52 days later (I4 + 52). Antibody responses are expressed as arbitrary units (AU) in relation to Tanzanian pooled serum (100). Each line represents a single subject. Differences were analyzed using Wilcoxon matched-pairs signed-rank test. Significant differences are indicated by * (*P* < 0.05) and ** (*P* < 0.01). CSP = circumsporozoite protein; LSA-1 = liver stage antigen 1; MSP-1 = merozoite surface protein 1.

First we addressed whether volunteers immunized with PfSPZ-CVac in group 1 had specific antibodies to PfCSP, PfLSA-1, or PfMSP-1 at 59 days after the third immunization and one day before CHMI. Antibodies to PfCSP were significantly increased compared with preimmunization (*P* = 0.03) with fold increases in titers ranging from 0.9 to 5.7 (median = 2.66). Five of 10 subjects showed a greater than 2-fold rise of anti-PfCSP antibody titers. Antibodies to PfLSA-1 and PfMSP-1 showed no significant increase (Figure 5A

). The increase in anti-PfCSP antibody titers was comparable in both PfSPZ-CVac groups (1 and 3) after the first two immunizations (*P* = 0.41).

The third immunization increased anti-PfCSP antibodies in 7/10 subjects in group 1, but in only one subject more than 2-fold (I3 − 1 versus I3 + 59; median fold increase with range = 1.26 [0.79–2.45]; *P* = 0.28). In contrast, after the fourth immunization anti-PfCSP antibodies were boosted in 8/8 subjects of group 3 and by at least 2-fold in three subjects (I4 − 1 versus I4 + 52; median fold increase with range = 1.43 [1.25–5.74]; *P* = 0.008; Figure 5B). Similarly, 7/8 subjects of group 3 had antibodies against PfSPZ by IFA after the fourth immunization (anti-PfSPZ titers ranged from 50 to 400, geometric mean = 110) in contrast to controls (anti-PfSPZ titer < 50). Although the proportion of anti-PfCSP responders increased in group 3 after the fourth dose, the magnitude of the antibody response remained similar to the post-third dose in group 1 (*P* = 0.24). These data suggest that four immunizations will increase the number of responders without further increasing the specific antibody titer. Antibodies against PfLSA-1 and PfMSP-1 did not significantly increase after the fourth immunization (data not shown).

In contrast to humoral responses, neither IFN-γ, CD107a nor granzyme B recall responses to PfRBC or PfSPZ, which were found to be indicative of parasite exposure previously,^26^,^29^ were induced after three PfSPZ-CVac immunizations in any of the T-cell subsets analyzed (vaccine group 1 versus control group 2, data not shown). Furthermore, even after the fourth PfSPZ-CVac immunization administered in group 3 there were still no measurable responses to PfRBC when compared with control group 4 or preimmunization (data not shown).

## Discussion

This first clinical study of PfSPZ-CVac showed that the ID immunization regimen of up to four doses of 7.5 × 10^4^ PfSPZ in healthy malaria-naive volunteers was safe and well tolerated, but did not confer detectible cellular immune responses and protection against a homologous CHMI. In contrast, a three-dose CPS protocol using PfSPZ-infected mosquitoes for immunization induces strong cellular responses and > 90% protection against CHMI as previously shown in a number of clinical trials.^5^,^7^,^26^ Dose-dependent sterile protection has been observed with 5/10 volunteers protected after PfSPZ-infected bites from a total of only 15 mosquitoes.^26^ In these CPS studies, transient parasitemia as detected by qPCR occurs in the majority of volunteers in particular after the first immunization.^5^ This reflects complete liver maturation followed by rapid chloroquine-mediated killing of blood-stage parasites. Under these conditions, the host's immune system is apparently sufficiently exposed to a critical parasite load and broad array of antigens for induction of protective pre-erythrocytic immune responses.^23^,^30^

In this study, we believe that insufficient numbers of PfSPZ migrated to and developed in the liver as supported by the weak humoral and absent cellular immune responses against Pf antigens and lack of sterile protection. CPS-induced humoral responses have been shown to correlate with the numbers of bites by PfSPZ-infected mosquitoes and thus with the degree of Pf-antigen exposure.^23^ Here, anti-PfCSP antibody responses after PfSPZ-CVac immunizations were comparable to responses after CPS immunizations,^23^ indicating exposure to adequate numbers of PfSPZ. However, anti-PfLSA-1 and anti-PfMSP-1 antibody responses were absent, reflecting very limited hepatocyte invasion and liver-stage development. This interpretation was further supported by the absence of cellular recall responses after PfSPZ-CVac immunizations, that is, parasite-specific IFN-γ, CD107a, or granzyme B responses, indicative of parasite exposure,^26^,^29^ and degranulation of CD4 T cells, previously shown to be associated with protection.^26^

In this trial, PfSPZ were administered ID by needle injection suggesting that the route of administration may influence outcome. In fact, murine data show that the route of administration of fresh and cryopreserved PfSPZ is a key determinant of successful liver infection; IV and IM injections result in significantly higher (∼50-fold and 2- to 3-fold, respectively) liver loads compared with ID and subcutaneous (SC) injections.^31^ Furthermore, Ploemen and others^31^ demonstrated that both IM and ID routes increase liver loads when using smaller volumes and injections at multiple sites. Such methods approach the way anopheline mosquitoes successfully administer SPZs: in some cases before a capillary is found the female mosquito probes the dermal skin several times, while injecting tiny volumes of sporozoite-containing saliva.^32^ In mice, compared with ID administration, IV inoculation of SPZs either radiation attenuated or in combination with chloroquine treatment increases parasite liver loads and augments cellular immune responses with higher protective efficacy against an infection with *Plasmodium berghei*.^33^

A series of clinical trials have been conducted to identify the lowest dose of PfSPZ Challenge that gives 100% infection with a prepatent period similar to CHMI studies with the standard regimen of five NF54-PfSPZ-infected mosquitoes, and a clear dose response. These trials show that IV or DVI administration is more efficient than IM, which is more efficient than the ID administration, all of which is in agreement with murine data; 100% infection has been achieved by IV and DVI,^10^ IM,^8^,^12^ and ID^34^ administration. However, a prepatent period of < 11.5 days and a dose response have only been achieved by IV/DVI and IM administration and have not been achieved by ID administration.^8^,^10^–^13^ As shown in murine models, the efficiency at which PfSPZs reach the liver as reflected by liver parasite loads is a direct result of the route of administration. This difference between IV and ID (or SC) administration has also been seen in human volunteers with the protective efficacy generated by radiation attenuated, aseptic, purified, and cryopreserved PfSPZ vaccine. Four to six doses of 1.35 × 10^5^ irradiated PfSPZ of PfSPZ vaccine administered SC or ID gave minimal immune responses and protection,^24^ whereas five doses of 1.35 × 10^5^ PfSPZ administered IV gave excellent immune responses and 100% protection.^35^ Thus, IV/DVI is the most effective and efficient method for PfSPZ administration.

The dose of 7.5 × 10^4^ PfSPZ-CVac administered ID in our study was well tolerated with remarkably few AEs, most of which were mild and occurred with the same frequency in controls, who received only NS. However, there was a cardiac SAE at 12 days after CHMI (59 days after the last dose of PfSPZ Challenge) and 2 days after initiation of curative treatment with atovaquone/proguanil in a subject of vaccine group 1. The subject was diagnosed as having acute myocarditis, but the pathophysiological basis for this acute myocarditis remains unclear and a definitive etiology could not be established.^18^

Interestingly, there was a discrepancy in the protection against CHMI by mosquito bite as assessed by thick smear and retrospective qPCR in two subjects of group 1, raising the possibility that early parasitemias could have been limited by residual chloroquine. Another finding suggesting an impact of chloroquine was the significantly prolonged Δ prepatency in control group 2 (Figure 3) compared with controls of recent previous CHMI studies. However, it is noteworthy that in a previous CHMI trial in which there were similar plasma concentrations (range < 5–14 μg/L for chloroquine and < 5 μg/L for monodesethylchloroquine),^5^ all five controls became thick smear positive between days 7 and 11 after CHMI. These plasma concentrations were deemed to be below the minimum therapeutic concentration in vivo based on literature (Rombo and others^28^: minimum = 30 μg/L), but also because identical blood-stage parasite multiplication kinetics were seen in control subjects compared with previous studies. This suggests that any residual chloroquine levels had no measurable parasiticidal effect.^36^,^37^ Thus, the reasons for the lack of development of parasite densities detectable by thick blood smear in the two subjects who were positive by qPCR are not understood.

In conclusion, this study shows that three to four doses of ID administered 7.5 × 10^4^ PfSPZ Challenge given with chloroquine, the PfSPZ-CVac regimen used in this trial, were well tolerated and safe, but did not protect against a homologous CHMI by mosquito bite. The lack of protection was almost certainly due to suboptimal parasite exposure, as supported by weak humoral responses and lack of T-cell responses previously shown to be associated with protection. A PfSPZ-CVac trial using DVI may result in high-level liver stage infection and protection.

## Supplementary Material

Supplemental Figure.

## Figures and Tables

**Table 1:** Trial summary table

	Group 1	Group 2	Group 3	Group 4
	*N* = 10	*N* = 5	*N* = 10 (*N* = 4 received CHMI)	*N* = 5 (*N* = 4 received CHMI)
Dose (number of PfSPZ)	3 × 75,000	0	3 × 75,000 (*N* = 2)	0
			4 × 75,000 (*N* = 8)	
Route of administration	ID	ID	ID	ID
Number of volunteers who became TS+	8	5	N/A	N/A
Listing of times to TS+ (days)	10.5, 10.5, 11, 12, 12, 14, 14.5, and 15	10.5, 10.5, 13.5, 14, and 16	N/A	N/A
Geometric mean time to TS+ (days)	13.7	12.7	N/A	N/A
Listing of parasite density by qPCR at time of TS+ (parasites/μL blood)	14, 26, 13, 40, 46, 66, 24, and 27	44, 120, 75, 32, and 1	N/A	N/A
Geometric mean parasite density by qPCR at time of TS+ (parasites/μL blood)	27.9	26.3	N/A	N/A
Number of volunteers who became qPCR+	10 (100%)	5 (100%)	4 (100%)	4 (100%)
Listing of times to qPCR+ (days)	7.0, 7.0, 7.0, 7.0, 7.0, 7.0, 9.0, 10.5, 10.5, and 10.5	7.0, 7.0, 7.0, 7.0, and 10.5	7.0, 10.5, 10.5, and 10.5	7.0, 7.0, 7.0, and 10.0
Geometric mean time to qPCR+ (days)	8.1	7.6	9.5	7.7
Listing of parasite densities by qPCR at time of qPCR+ (parasites/μL blood)	0.08, 1.01, 0.09, 0.13, 0.25, 0.07, 0.08, 0.05, 0.05, and 0.08	1,02, 0.47, 0.90, 0.13, and 1.78	0.74, 1.40, 0.08, and 0.06	0.86, 0.36, 0.97, and 0.52
Geometric mean parasite density by qPCR at time of qPCR+ (parasites/μL blood)	0.11	0.63	0.27	0.63

CHMI = controlled human malaria infection; ID = intradermal; N/A = not applicable; PfSPZ = *Plasmodium falciparum* sporozoite; qPCR+ = quantitative real-time polymerase chain reaction positive; TS+ = thick smear positive.

**Table 2:** AEs during immunizations #1–4

Adverse event*	PfSPZ-CVac groups 1† (*N* = 10) and 3 (*N* = 2†/*N* = 8‡)			Control groups 2† (*N* = 5) and 4‡ (*N* = 5)		
	No. of volunteers	Mean duration ± SD (days)	Occurrence after injections (days)	No. of volunteers	Mean duration ± SD (days)	Occurrence after injections (days)
Abdominal pain	2	0.9 ± 1.0	−6, 25	2	0.1 ± 0.02	1–10
Chills	2	1.1 ± 1.2	2–4, 4	N/A	N/A	N/A
Diarrhea	1	0.6	20	N/A	N/A	N/A
Headache	6	0.2 ± 0.2	−2 to 33	3	0.5 ± 0.6	1–20
Nausea	4	0.4 ± 0.6	−6 to 27	1	0.1 ± 0.1	20
Vomiting	2	0.02 ± 0.0	−1, 6	N/A	N/A	N/A
Any	11	0.3 ± 0.5	N/A	6	0.3 ± 0.4	N/A

AE = adverse events; N/A = not applicable; PfSPZ-CVac = *Plasmodium falciparum* sporozoite chemoprophylaxis vaccine; SD = standard deviation.

* Subjects could have more than one AE. Only solicited AEs that were possibly or probably related to the study are listed. Solicited AEs were fever, headache, malaise, fatigue, dizziness, myalgia, arthralgia, nausea, vomiting, chills, diarrhea, abdominal pain, chest pain, palpitations, and shortness of breath.

† Total of three PfSPZ or normal saline immunizations.

‡ Total of four PfSPZ or normal saline immunizations.

**Table 3:** AEs after mosquito CHMI

CHMI #1	PfSPZ-CVac group 1 (*N* = 10)		Control group 2 (*N* = 5)	
AE*	No. of volunteers	Mean duration ± SD (days)	No. of volunteers	Mean duration ± SD (days)
Abdominal pain	2	0.5 ± 0.1	2	0.1 ± 0.1
Chest pain, unspecified	1	0.0	0	N/A
Chills	6	0.2 ± 0.1	3	0.4 ± 0.2
Diarrhea	0	N/A	1	0.1 ± 0.0
Dizziness	1	1.2	2	0.1 ± 0.0
Fatigue	2	4.0 ± 5.2	1	0.4 ± 0.3
Fever	7	0.3 ± 0.3	4	0.6 ± 0.4
Headache	8	0.5 ± 0.4	5	0.9 ± 1.1
Malaise	1	0.3	1	0.5
Myalgia	2	1.2 ± 0.7	3	1.2 ± 0.7
Nausea	5	0.3 ± 0.3	3	0.3 ± 0.4
Vomiting	2	0.0 ± 0.0	1	0.2
Any	10	0.6 ± 1.2	5	0.6 ± 0.7
Grade 3 AE				
Fever	3	0.2 ± 0.1	1	0.5
Headache	1	0.4	0	N/A
Nausea	0	N/A	1	0.3
Vomiting	2	0.0 ± 0.0	1	0.2
Any	4	0.1 ± 0.2	2	0.3 ± 0.2
CHMI #2	PfSPZ-CVac group 3 (*N* = 4)		Control group 4 (*N* = 4)	
AE	No. of volunteers	Mean duration ± SD (days)	No. of volunteers	Mean duration ± SD (days)
Abdominal pain	0	N/A	2	0.3 ± 0.2
Chills	1	0.3	2	0.0 ± 0.0
Dizziness	0	N/A	1	0.1
Fever	1	0.6	2	0.8 ± 1.0
Headache	3	0.4 ± 0.5	3	2.4 ± 3.2
Malaise	1	2.2	0	N/A
Myalgia	1	0.6 ± 0.6	1	1.5
Nausea	3	0.3 ± 0.2	2	0.2 ± 0.1
Vomiting	1	0.0	0	N/A
Any	3	0.5 ± 0.6	4	0.9 ± 1.9
Grade 3 AE				
Fever	0	N/A	1	0.4
Headache	0	N/A	1	0.1
Vomiting	1	0.0	0	N/A
Any	1	0.0	1	0.3 ± 0.2

AE = adverse event; CHMI = controlled human malaria infection; N/A = not applicable; PfSPZ-CVac = *Plasmodium falciparum* sporozoite chemoprophylaxis vaccine; SD = standard deviation.

* Subjects could have more than one AE. Only solicited adverse events that were possibly or probably related to the study are listed.
