# Supplementary material for: Safety, Immunogenicity, and Protective Efficacy of Intradermal Immunization with Aseptic, Purified, Cryopreserved Plasmodium falciparum Sporozoites in Volunteers under Chloroquine Prophylaxis: A Randomized Controlled Trial
Source: Am J Trop Med Hyg. 2016 Mar 2;94(3):663–73. doi: 10.4269/ajtmh.15-0621 (PMC4775905; doi:10.4269/ajtmh.15-0621)
Supplement: Supplementary file 1 [file SD12.pdf]

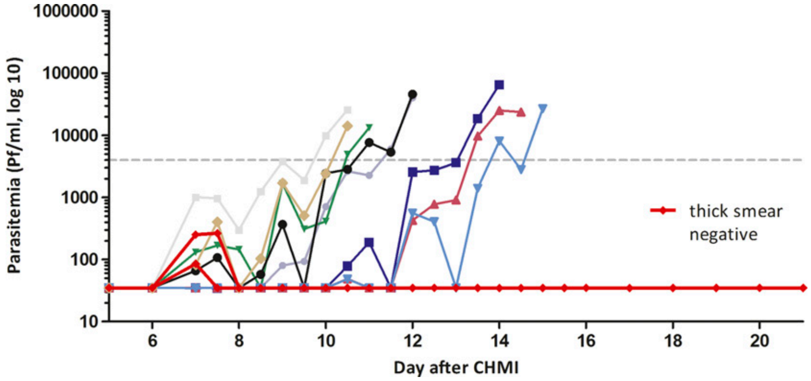

Supplemental Figure 1. Parasite density in vaccine group 1 after controlled human malaria infection #1. Individual parasite density curves of vaccine group 1 subjects ($N = 10$) measured by qPCR are shown up to day of treatment, based on diagnosis by thick smear. The grey dotted line indicates the average parasite detection limit for microscopy. Two subjects remained thick smear negative through day 21 post-CHMI.
